# Maintained Hydration Status After a 24-h Winter Mountain Running Race Under Extremely Cold Conditions

**DOI:** 10.3389/fphys.2018.01959

**Published:** 2019-01-11

**Authors:** Daniela Chlíbková, Pantelis T. Nikolaidis, Thomas Rosemann, Beat Knechtle, Josef Bednář

**Affiliations:** ^1^Centre of Sports Activities, Brno University of Technology, Brno, Czechia; ^2^Institute of Primary Care, University of Zurich, Zurich, Switzerland; ^3^Exercise Physiology Laboratory, Nikaia, Greece; ^4^Medbase St. Gallen Am Vadianplatz, St. Gallen, Switzerland; ^5^Faculty of Mechanical Engineering, Brno University of Technology, Brno, Czechia

**Keywords:** extreme weather conditions, winter, ultra-running, fluid intake, plasma sodium

## Abstract

**Background:** To date, no study has examined the hydration status of runners competing in a 24-h winter race under extremely cold environmental conditions. Therefore, the aim was to examine the effect of a 24-h race under an average temperature of -14.3°C on hydration status.

**Methods:** Blood and urine parameters and body mass (BM) were assessed in 20 finishers (women, *n* = 6; men, *n* = 14) pre- and post-race.

**Results:** Five (25%) ultra-runners had lower pre-race plasma sodium [Na^+^] and 11 (52%) had higher pre-race plasma potassium [K^+^] values than the reference ranges. Post-race plasma [Na^+^], plasma osmolality, urine osmolality and urine specific gravity remained stable (*p* > 0.05). The estimated fluid intake did not differ (*p* > 0.05) between women (0.30 ± 0.06 L/h) and men (0.46 ± 0.21 L/h). Runners with a higher number of completed ultra-marathons (*r* = -0.50, *p* = 0.024) and higher number of training kilometers (*r* = -0.68, *p* = 0.001) drank less than those with lower running experience. Pre-race and post-race plasma [Na^+^] were related to plasma osmolality (*r* = 0.65, *p* = 0.002, *r* = 0.69, *p* < 0.001, respectively) post-race, but not to fluid intake (*p* > 0.05). BM significantly decreased post-race (*p* = 0.002) and was not related to plasma [Na^+^] or fluid intake (*p* > 0.05). Post-race hematocrit and plasma [K^+^] decreased (*p* < 0.001) and transtubular potassium gradient increased (*p* = 0.008). Higher pre-race plasma [K^+^] was related to higher plasma [K^+^] loss post-race (*p* = -0.85, *p* < 0.001).

**Conclusion:** Hydration status remained stable despite the extremely cold winter weather conditions. Overall fluid intake was probably sufficient to replenish the hydration needs of 24-h runners. Current recommendations may be too high for athletes competing in extremely cold conditions.

## Introduction

Events such as 24-h ultra-marathons are accompanied by a multitude of stressors including physical exertion, sleep deprivation and/or environmental extremes and there is clearly a high risk of hyperhydration during a race (Costa et al., 2014). Exercise-associated hyponatremia was demonstrated in one (8%) 24-h ultra-runner (temperatures ranging between 10 and 18°C) and six (12%) 24-h mountain bikers (temperatures ranging between 6 and 30°C) during summer races in our recent study (Chlíbková et al., 2016).

To date, there are limited studies on hydration status in ultra-endurance athletes racing in cold weather conditions (Stuempfle et al., 2002, 2003; Paulin et al., 2015). Sleep deprivation and low air temperature reduce body core temperature to a greater extent (Castellani et al., 2003). Even a mild decrease in total body water and cold diuresis (Lennquist et al., 1974) can cause dehydration since cold attenuates thirst (Stuempfle et al., 2002) and diuresis exacerbates body water loss (Dann et al., 1990). By contrast, only one study to date has demonstrated that athletes in a winter (-8 to 4°C) ultra-endurance event (cyclists and runners) showed exercise-associated hyponatremia (Stuempfle et al., 2002) due to excessive fluid consumption and/or inadequate sodium intake. According to Stuempfle (2010), winter athletes can overdrink for behavioral reasons and/or develop problems due to inappropriate arginine vasopressin secretion during prolonged exercise. Multiple risk factors have been asocciated with exercise-associated hyponatremia, including extremely hot or cold environmental conditions (Stuempfle et al., 2002; Stuempfle, 2010; Hew-Butler et al., 2015). It is worth noting that seven athletes developed exercise-associated hyponatremia during a 161-km running ultra-endurance race in Alaska in 2000 (Stuempfle et al., 2002); however, no athlete developed exercise-associated hyponatremia during the same race in 2001 (Stuempfle et al., 2003). Exercise-associated hyponatremia is probably more common in hot environments, since people lose a lot of body fluid due to sweating and evaporation in order to cool the body and there is a higher chance of overdrinking. When the temperature is very low, the prevalence of exercise-associated hyponatremia in ultra-marathoners seems to decrease (Wagner et al., 2012). It appears that ultra-runners in winter conditions should be less prone to exercise-associated hyponatremia, as cold environment causes hypohydration due to decreased thirst and diuresis (Stuempfle, 2010).

To the best of our knowledge, no previous studies have investigated hydration status in athletes racing for 24 h under extremely cold winter conditions with an average temperature of -14.3°C. Furthermore, the risk of exercise-associated hyponatremia in very cold weather conditions has not been thoroughly investigated yet. With this in mind, the aim of this pilot observational study was to quantify the hydration status in female and male ultra-marathoners competing in a 24-h winter ultra-marathon.

## Materials and Methods

The study received ethical approval from the institutional review boards of the Centre of Sports Activities at Brno University of Technology and the Institute of Experimental Biology at Masaryk University in Brno, Czechia, that conforms with the 2008 Helsinki declaration for human research ethics.

### Participants

Race participants were notified of the study approximately 2 months and again 1 week before the race start via an e-mail sent by the race organizers. They were informed about the planned investigation and that their participation in the study is voluntary. No inclusion/exclusion criteria were used, with the exception that the participants had to run (walk) continuously and for the whole 24 h of the race without any sleeping rests. All volunteers provided a written informed consent and filled out a pre-race on-line questionnaire requesting information on previous running experience. Overall, 632 participants (506 men and 124 women) started in the 24-h winter running race and 606 runners (95.9%) successfully finished the race (486 men and 120 women). Twenty-one amateur ultra-runners volunteered to participate in the study. For the comparison of participants and non-participants in our study, we used data provided by the race organizers in the final results on their website. There were no differences in sex, age, or performance (speed, number of completed kilometers during the 24-h race) between the participants and non-participants in the study (*p* > 0.05).

### The Race

The 24-h winter running race entitled Adidas 24-h open championship of the Czechia in winter mountain ultra-marathon of individuals on the Lysá Mountain’ started on January 25, 2014, at 10:58 a.m. and finished on January 26, 2014, at 10:58 a.m. in Ostravice, Czechia. The lowest temperature was -20.6°C and the highest was -7.9°C. The temperature at the top of Lysá Mountain (1,323 m) was around -19°C. In addition, there was a gusty wind which reduced the air temperature to the wind chill values of -28.9°C (January 25) and -18.3°C (January 26). The average humidity was 88.5%, precipitation was 0.3 mm and the snow depth was 1 cm. One lap measured 11.4 km with 764 m of elevation in very rugged terrain. The aim of the race is to achieve the highest number of laps, i.e., repeated ascents and descents of the summit of Lysá Mountain within 24 h. The strategy regarding food, rest, and sleep was at the discretion of each participant. Measurement of the race was carried out electronically using chips and only completed circuits were taken into account. There was one aid station located at the start/finish of the circuit which offered water, isotonic drinks, tea, soup, fruit, and cereals. Runners could also use their own refreshments.

### Measurement and Calculations

During pre-race sample collection, participants were educated about the volumes of cups offered at the aid station and instructed to recall fluid intake at the finish during post-race measurements. During the race, assistants marked the number of cups consumed by the runners at the aid station. Fluids that were part of a meal or snack were not recorded. The fluid intake was also estimated and recorded by each athlete or by their support team on a recording sheet. Final fluid intake was estimated based on the reports and additional information provided by assistants at the aid station. The event website did not provide the athletes with any special advice on what and how much they should drink during the race.

Pre-race testing was performed on the starting day from 8:00 to 11:00 a.m. Post-race measurements were taken immediately after the race and were finished within 2 h when all the participants reached the start/finish of the race and some of them were finally able to submit urine samples due to problems with antidiuresis. The pre- and post-race measurement procedures were identical. Body mass (BM) was measured using a calibrated commercial scale (Tanita BC-351, Tanita Corporation of America, Inc.) to the nearest 0.1 kg. Blood samples were drawn from an antecubital vein within 5 min of finishing. Standardization of the sitting position prior to blood collection was respected since postural changes can influence blood volume and hematocrit (Hct). One Sarstedt S-Monovette (plasma gel, 7.5 mL) for chemical analysis was cooled and one Sarstedt S-Monovette (EDTA, 2.7 mL) for hematological analysis was kept at 15–25°C. Both were sent to the laboratory and analyzed within 6 h. Hct was determined using Sysmex XE 2100 hematology analyzer (Sysmex Corporation, Japan). Plasma [Na^+^] and plasma [K^+^] were determined on biochemical analyzer Modular SWA, Module P + ISE (Hitachi High Technologies Corporation, Japan; Roche Diagnostics). Plasma osmolality (Posm) was determined using Arkray Osmotation (Arkray Factory, Inc., Japan). Urine samples were collected in Sarstedt monovett for urine (10 mL) and sent to the laboratory. Urinary [Na^+^] and [K^+^] were determined using biochemical analyzer Modular SWA, Module P + ISE (Hitachi High Technologies Corporation, Japan, Roche Diagnostics), urinary specific gravity (Usg) was determined using Au Max-4030 (Arkray Factory, Inc., Japan), and urine osmolality (Uosm) was determined using Arkray Osmostation OM-650 (Arkray Factory, Inc., Japan). Individual post-race Usg samples in the range of 1.013–1.029 g/mL were considered normal, 1.030 g/mL or higher indicated significant dehydration and Usg below 1.012 g/mL was regarded as hyperhydration (Armstrong et al., 1998). Transtubular potassium gradient (TTKG) was calculated using the formula (potassium_urine_ × osmolality_serum_)/(potassium_serum_ × osmolality_urine_) (West et al., 1986).

For the evaluation of blood parameters, laboratory reference values for adults (Kratz et al., 2004) were used (male Hct 41.0–53.0%, female Hct 36.0–46.0%; plasma [Na^+^] 136–145 mmol/L; plasma [K^+^] 3.5–5.0 mmol/L). Hyperkalemia was classified according to serum [K^+^] as mild (5.5–6.5 mmol/L), moderate (6.5–7.5 mmol/L) or severe (>7.5 mmol/L) hyperkalemia (Lehnhardt and Kemper, 2011). However, strenuous exercise may have a profound effect on laboratory parameters (Kratz et al., 2002). Therefore, we also used a table of modified reference ranges for basic biochemical and hematological laboratory parameters derived from marathon runners (Kratz et al., 2002), which are identical for both sexes (pre-race Hct 39–49%, post-race Hct 38–48%; pre-race plasma [Na^+^] 139–146 mmol/L, post-race plasma [Na^+^] 134–149 mmol/L; pre-race plasma [K^+^] 3.7–5.3 mmol/L, post-race plasma [K^+^] 3.5–5.5 mmol/L). The results were also compared with reference ranges for endurance elite athletes (Sharpe et al., 2002) (male Hct values of 34.3–45.0%, female values of 38.8–49.6%).

### Statistical Analysis

Descriptive statistics (mean, standard deviation) were calculated for pre- and post-race values and absolute and percentage changes of blood, urinary, and BM parameters. Normal distribution was verified by Anderson-Darling’s test for normality. The data was often not normally distributed, and even if it passed the normality test, there was a high risk of type II error due to the small size of the groups. Therefore, non-parametric tests were used. Comparisons between the male and female groups (mean values of fluid intake, age, years as an active runner, number of completed ultra-marathons, mean weekly total training volume and training volume in running, number of training kilometers in the year prior to this race, longest run in the week prior to the race, average running speed and the number of completed kilometers in the present 24-h race) were made using unpaired non-parametric two-sample Wilcoxon test (also known as Wilcoxon rank sum test or Mann–Whitney test). Pair data, especially the mean pre- and post-race values within each group were analyzed using paired one sample Wilcoxon rank sum test. Blood and urine data were analyzed separately for males and females and two-sample Wilcoxon test was used for between-group comparisons of continuous data. The dependence between the individual characteristics were tested using the Spearman’s correlation coefficient. The data was processed using MINITAB 17 statistical software. Statistical significance was set at *p* < 0.05.

## Results

A total of 20 (95.2%) athletes (14 men and 6 women) out of those who volunteered for the study finished the race with a full data set. One athlete did not finish due to frostbite and hypothermia. Pre-race experience, training variables, average running speed and the number of completed kilometers of male and female finishers are shown in Table 1. No differences were found between male and female finishers in age, years as an active runner, number of completed ultra-marathons, mean weekly total training volume and training volume in running, number of training kilometers in the year prior to this race, longest run in the week prior to the race, average running speed and the number of completed kilometers in the present 24-h race (*p* > 0.05) (Table 1). The only difference was in the average speed during the 24-h race (*p* = 0.012) where male runners were faster than female runners.

**Table 1:** Pre-race experience, training, average speed and completed kilometers of male and female finishers (*n* = 14 and *n* = 6, respectively).

	Men (*n* = 14)	Women (*n* = 6)	*p*-Value
Age (years)	30.0 ± 10.7	37.5 ± 12.9	0.201
Experience as an active runner (years)	8.0 ± 7.8	8.0 ± 7.6	0.621
Finished ultra-marathons (n)	4.4 ± 3.1	5.5 ± 3.9	0.592
Mean weekly total training volume (h)^†^	7.8 ± 4.1	9.0 ± 5.9	0.964
Mean weekly training volume in running (h)^†^	6.0 ± 2.0	7.7 ± 4.2	0.564
Training distance in the previous year (km)	1062. ± 629	1333.3 ± 455.0	0.458
Longest run in the week prior to the race (km)	32.5 ± 20.0	37.1 ± 15.6	0.509
Average race speed (km/h)	4.8 ± 1.2	3.3 ± 1.1	0.012^∗^
Number of completed kilometers in 24 h (km)	83.0 ± 27.4	68.5 ± 24.4	0.248


*^†^During the 3 months before the event. Data are expressed as mean ± SD. ^∗^*p* < 0.05.*

### BM, Posm, Plasma [Na^+^], and Fluid Intake

**FIGURE 1:**
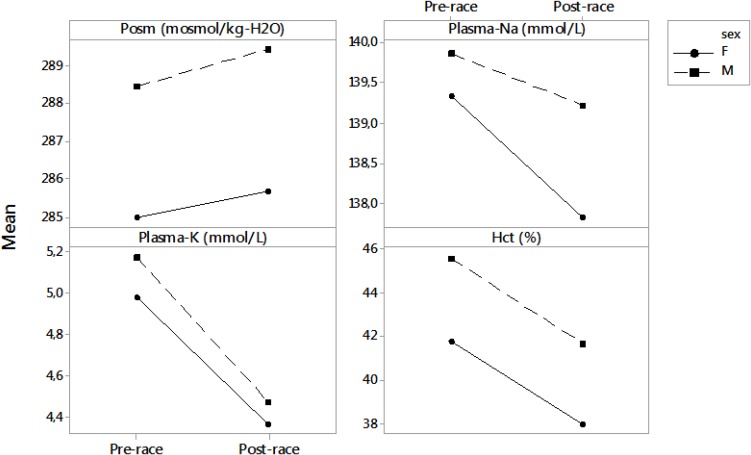
Differences between pre- and post-race blood parameters in male and female runners. Results are presented as mean. F, female; M, male; Posm, plasma osmolality; Plasma-Na, plasma sodium; Plasma-K, plasma potassium; Hct, hematocrit.

The average BM decreased significantly post-race (*p* = 0.002). BM change was not related to plasma [Na^+^] change post-race (*p* > 0.05). According to the modified reference ranges by Kratz et al. (2002), five runners (25%), three men and two women, showed lower pre-race plasma [Na^+^] values (in the range of 137–138 mmol/L). Post-race plasma [Na^+^] and plasma osmolality remained stable (*p* > 0.05) (Table 2) in both men and women (Figure 1). All runners showed post-race plasma [Na^+^] within the reference laboratory ranges (Kratz et al., 2004), as well as within the modified reference ranges (Kratz et al., 2002). Pre-race and post-race plasma [Na^+^] were related to Posm (*r* = 0.65, *p* = 0.002, *r* = 0.69, *p* < 0.001, respectively) post-race, but not to fluid intake (*p* > 0.05). Fluid intake during the race was on average 0.42 (0.19) L/h, ranging from 0.20 to 1 L/h. Female runners drank 0.30 (0.06) L/h, ranging from 0.20 to 1 L/h, while male runners drank 0.46 (0.21) L/h, ranging from 0.20 to 1 L/h, without significant differences between the sexes (*p* > 0.05). Fluid intake was negatively associated with the number of finished ultra-marathons (*r* = -0.50, *p* = 0.024) and the number of training kilometers (*r* = -0.68, *p* = 0.001). Post-race plasma [Na^+^] and/or post-race BM were not associated with fluid intake (*p* > 0.05).

**Table 2:** Blood and urine parameters, body mass changes (*n* = 20).

	Pre-race	Post-race	Absolute change	Percentage change	*P*-value
Hematocrit (%)	44.5 ± 2.9	40.6 ± 2.7	-3.9 ± 2.2	-8.7 ± 4.7	<0.001^∗^
Plasma sodium (mmol/L)	139.7 ± 1.8	138.8 ± 2.5	-0.9 ± 2.1	-0.6 ± 1.5	0.069
Plasma potassium (mmol/L)	5.1 ± 0.6	4.4 ± 0.3	-0.7 ± 0.5	-13.3 ± 9.0	<0.001^∗^
Plasma osmolality (mOsmol/kg H_2_O)	287.4 ± 4.7	288.3 ± 6.3	0.9 ± 5.1	0.3 ± 1.8	0.514
Urine sodium (mmol/L)	107.4 ± 72.7	63.4 ± 30.4	-44.0 ± 78.3	-5.6 ± 72.8	0.048^∗^
Urine potassium (mmol/L)	36.6 ± 22.9	51.1 ± 23.5	14.5 ± 31.2	119.7 ± 206.3	0.048^∗^
Urine osmolality (mOsmol/kg H_2_O)	589.2 ± 261.3	707.1 ± 256.3	117.9 ± 305.5	63.3 ± 136.0	0.151
Urine specific gravity (g/mL)	1.022 ± 0.007	1.024 ± 0.006	0.003 ± 0.008	0.3 ± 1.0	0.121
Transtubular potassium gradient	3.6 ± 1.5	4.8 ± 1.8	1.2 ± 1.6	51.9 ± 75.5	0.008^∗^
Body mass (kg)	76.0 ± 10.3	75.2 ± 9.9	-0.8 ± 1.1	-0.9 ± 1.4	0.002^∗^


*Data are expressed as mean ± SD. ^∗^*p* < 0.05.*

### Hematocrit

Pre-race Hct was in the range of 40.2–48.9% in men and 38.1–44.6% in women. Post-race Hct reached 37.3–44.5% in men and 35.0–41.1% in women. The decrease in Hct was significant (*p* < 0.001) (Table 2). All runners showed pre-race Hct values within the reference ranges (Kratz et al., 2004), except for one male runner with Hct of 40.2%, which is, however, still acceptable according to Kratz et al. (2002) or Sharpe et al. (2002). Four (20%) runners (one man and three women) showed lower post-race values than the values provided by Kratz et al. (2002). Post-race Hct was not associated with plasma [Na^+^], Posm or fluid intake (*p* > 0.05). Differences between pre- and post-race blood parameters in male and female runners are noted in Figure 1.

### Plasma [K^+^]

**FIGURE 2:**
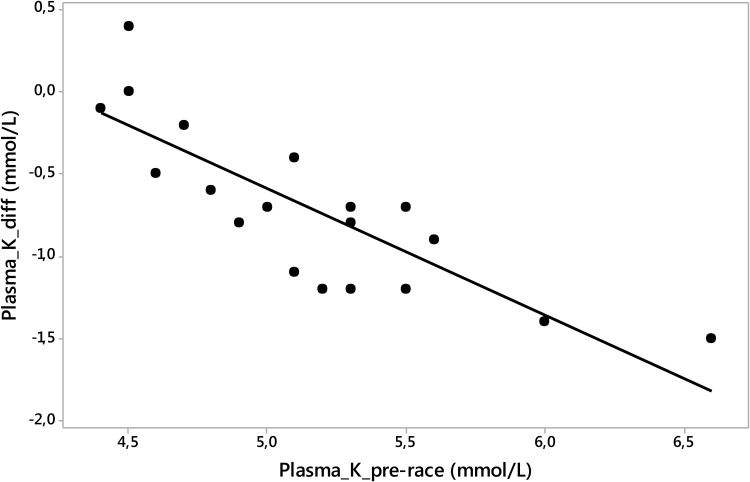
Pre-race plasma [K^+^] was negatively associated with plasma [K^+^] change post-race (*p* = –0.85, *p* < 0.001). Plasma_K_pre-race, plasma potassium pre-race; plasma_K_diff, plasma potassium difference post- minus pre- race value.

According to the laboratory reference values for adults (Kratz et al., 2004), 11 runners (55%), nine men and two women, showed higher pre-race plasma [K^+^] (ranging from 5.10 to 6.60 mmol/L). Mild pre-race hyperkalemia occurred in three (14%) males and one (5%) female runner and one (5%) man showed moderate hyperkalemia (Lehnhardt and Kemper, 2011) of 6.60 mmol/L post-race. Post-race plasma [K^+^] decreased significantly (Table 2); however, the post-race plasma [K^+^] values were within the modified reference range proposed by Kratz et al. (2002) for marathoners immediately after a marathon and also within the laboratory reference values for adults according to the study by Kratz et al. (2004). Pre-race plasma [K^+^] was negatively associated with plasma [K^+^] change post-race (*p* = -0.85, *p* < 0.001) (Figure 2). Differences between pre- and post-race blood parameters in male and female runners are listed in Figure 1.

### Usg, Uosm, Urine [Na^+^] and [K^+^], TTKG

**FIGURE 3:**
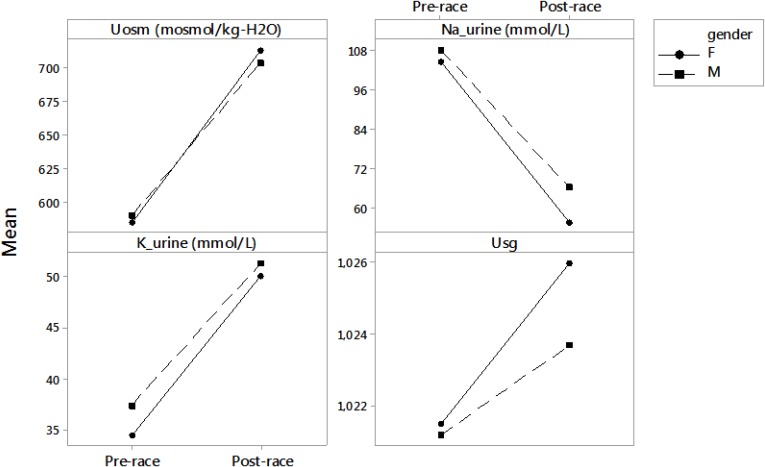
Differences between pre- and post-race urine parameters in male and female runners. Results are presented as mean. F, female; M, male; Uosm, urine osmolality; Na-urine, urine sodium; K-urine, urine potassium; Usg, urine specific gravity.

Usg and Uosm remained stable after the race (*p* > 0.05) (Table 2). TTKG significantly increased (*p* < 0.001) (Table 2). Urine [Na^+^] decreased (*p* = 0.048) and urine [K^+^] increased (*p* = 0.048); however, it was on the border of significance (Table 2). Differences between post- and pre-race urine parameters in male and female runners are listed in Figure 3. Post-race Usg values ranged from 1.015 to 1.030 g/mL. According to Armstrong et al. (1998), all finishers were euhydrated.

## Discussion

The aim of the study was to quantify hydration status in 24-h runners under extremely cold winter environmental conditions. Post-race plasma [Na^+^], Posm, Uosm, and Usg remained stable, while BM decreased during the race. The main finding was that hydration status remained stable.

### BM, Plasma [Na^+^], Posm, and Fluid Intake

Body mass decreased post-race, however, BM decreases during 24-h ultra-marathons are common and were noted by Fellmann et al. (1988), Kao et al. (2008), Knechtle et al. (2011), and Costa et al. (2014). Moreover, reductions in BM could also be expected due to respiratory water loss which is greater in the cold and due to diuresis, which is also a possible route for fluid losses (Nimmo, 2004). The most important finding was that plasma [Na^+^] and Posm remained stable after the race. Similarly, Posm did not change in the study of 24-h ultra-runners described by Costa et al. (2014) or in our previous study of 24-h ultra-runners and 24-h mountain bikers Chlíbková et al., 2014, where plasma [Na^+^] did not significantly change in both runners and mountain bikers. Moreover, post-race plasma [Na^+^] remained within the normal range in the present runners (Kratz et al., 2002).

By contrast, plasma [Na^+^] significantly decreased in winter cold races in Alaska (Stuempfle et al., 2002, 2003) with and without the prevalence of exercise-associated hyponatremia (-4.1 and -2.4 mmol/L, respectively). Stuempfle et al. (2003) suggested that the decrease in serum [Na^+^] in their study was not caused by the temperature, but by fluid overload. In the cold winter race (-8 to 4°C), 16 (44%) subjects developed exercise-associated hyponatremia, with a fluid intake of 0.5 L/h in the hyponatremic group and 0.4 L/h in the normonatremic group (Stuempfle et al., 2002). In the same race in a colder environment (-14 to -2°C) and with lower fluid intake (0.30 L/h), no exercise-associated hyponatremia occured, even though plasma [Na^+^] also decreased significantly due to overall fluid overload according to Stuempfle et al. (2003). In the current study, plasma [Na^+^] was not related to BM or fluid intake. The estimated average fluid intake during the current 24-h winter race was 0.4 L/h similarly as in the study of 24-h runners in summer conditions (0–20°C) by Costa et al. (2014), where the average water intake was 0.38 L/h. The fluid intake of 0.58 L/h in a 24-h running race during summer, but in cold conditions (10–18°C and heavy rain showers) in our previous study of 24-h runners (Chlíbková et al., 2014) seems to be higher in comparison with the results of the present study, however, plasma [Na^+^] and Posm remained stable in both studies.

The next finding was that we did not find any significant difference in fluid intake between male and female runners; moreover, female runners drank only 0.3 L/h. Nevertheless, disorders of fluid and electrolyte metabolism are frequently found in female endurance athletes (Speedy et al., 1999). Moreover, sex hormones may influence fluid balance parameters during the luteal phase of the menstrual cycle (Tam et al., 2009). Female marathoners drank more fluids in proportion to their body size, were significantly lighter and ran slower than male runners according to Hew (2005). Notwithstanding, the present female runners showed stable plasma [Na^+^], Posm and Usg post-race, therefore, we did not presume fluid overload.

The decrease in plasma [Na^+^] in the extremely cold winter conditions was non-significant in the current study; however, another finding was that a quarter of the present runners showed lower pre-race plasma [Na^+^] values than the modified reference ranges derived from marathon runners (Kratz et al., 2002). Lower plasma [Na^+^] pre-race appeared to be a risk factor for lower plasma [Na^+^] in a study of non-elite marathon runners (Chorley et al., 2007) and could mean that the athletes drank beyond their level of thirst before the race. The present runners with lower pre-race plasma [Na^+^] levels showed lower Posm concentrations post-race. As hypohydration does not increase hypothermia (O’Brien et al., 1998) or frostbites (O’Brien and Montain, 2003) and in winter athletes it is unlikely that hypohydration would increase cold weather related injuries (O’Brien et al., 1998; O’Brien and Montain, 2003); overhydration and the risk of exercise-associated hyponatremia is by contrast significant (Stuempfle, 2010). Nearly 86% of the post-race hyponatremic and 68% of the normonatremic ultra-athletes in our previous study (Chlíbková et al., 2016) drank greater volumes before the race than their thirst dictated based on the individual pre-race Usg values. Therefore, we do not recommend drinking beyond thirst also before the race.

We confirmed that post-race plasma [Na^+^] was related to post-race plasma osmolality. Fortunately, both variables remained stable. The recommendations for fluid consumption during the race of 0.5 to 1.0 L/h (Speedy et al., 2000) may be too high for ultra-runners competing in extremely cold conditions. Moreover, an interesting fact was that runners with a higher number of completed ultra-marathons and higher number of training kilometers drank less than those with lower running experience in the present study. Event inexperience and inadequate training are one of the most important risk factors for the development of asymptomatic and symptomatic exercise-associated hyponatremia (Hew-Butler et al., 2015). Current fluid intake was probably appropriate to replenish the hydration needs of 24-h runners and therefore fluid overload was not observed.

### Plasma [K^+^]

Although plasma [K^+^] decreased significantly, post-race values were within the laboratory reference ranges (Kratz et al., 2004), as well as within the modified reference ranges (Kratz et al., 2002). A rapid decrease in serum [K^+^] levels was shown immediately after exercise (Stanbie et al., 1982; Gerth et al., 2002) and also 6 h after a 100-km running race (Gerth et al., 2002). A significant decrease in plasma [K^+^] and Hct, but stable plasma [Na^+^] and Posm was demonstrated in extreme mountain ultra-marathoners post-race in the study by Zanchi et al. (2017). Plasma [K^+^] decreased, whereas plasma [Na^+^] remained stable in male and female runners also in our recent study of 24-h runners in a summer race (Chlíbková et al., 2014). Furthermore, seven (58%) of the 24-h runners in the above-mentioned study developed pre-race hyperkalemia. Similarly, mild to moderate pre-race hyperkalemia (Lehnhardt and Kemper, 2011) was observed in more than half of the present 24-h runners. The presence of mild pre-race hyperkalemia might have been caused by excessive [K^+^] intake or pre-race ingestion of non-steroidal anti-inflammatory drugs (Lehnhardt and Kemper, 2011); however, we did not inquire about this information. Higher pre-race plasma [K^+^] concentrations were associated with greater decreases in plasma [K^+^] post-race and high pre-race plasma [K^+^] concentrations could probably stimulate the secretion of aldosterone (Verbalis, 2003). The maintenance of water and solute homeostasis could be regulated by aldosterone, similarly as in the study by Tam et al. (2009), however, this hormone was not measured in this study.

The reason for the negative balance of plasma [K^+^] during the present 24-h race could also be energy, or fluid and energy restriction (James and Shirreffs, 2013). Low temperatures can also increase carbohydrate metabolism (Jeukendrup, 2004). The depletion of glycogen stores in working muscles and in the liver may be associated with blood [K^+^] loss (Hultman, 1967). Plasma [K^+^] is closely linked to blood sugar (glucose) and if blood sugar levels drop, plasma [K^+^] levels decrease. We presume that a substantial energy deficit occurred, despite *ad libitum* intake, similarly as in the study of 24-h ultra-runners by Costa et al. (2014), which could cause the decrease in plasma [K^+^]. Most of the 24-h ultra-runners in the above-mentioned study probably failed to meet the recommendations for sufficient carbohydrate intake corresponding to the strain induced by the 24-h race. In summary, mild [K^+^] losess are not dangerous for human organism due to the potassium reservoir function of various tissues (Lijnen et al., 1989). We assume that slow running pace could allow the renal blood flow to recover, similarly as in the mountain run (Page et al., 2007), and therefore hydration status remained stable.

### Hematocrit

A reduction in Hct post-race was significant in the present runners, so hyperhydration may be of greater concern than hypohydration. Similarly, prolonged strenuous endurance race in cold conditions led to a decrease in Hct due to hemodilution by retention of body water in the studies by Stuempfle et al. (2002, 2003). Fellmann et al. (1999) observed an increase of plasma volume during a 7-day 620-km race in moderate conditions (0–20°C) and assumed that prolonged exercise induced chronic hyperhydration at both extracellular and intracellular levels. Fellmann et al. (1988) concluded that plasma [Na^+^] retention is a major factor for the increased plasma volume. The hemodilution might be due to [Na^+^] retention and [K^+^] secretion due to increased aldosterone activity (Fellmann et al., 1999) as well as due to arginine vasopressin (AVP) increase (Fellmann et al., 1988). However, we did not measure aldosterone and AVP in this study. Nevertheless, Hct change after the race was not associated with stable plasma [Na^+^] and/or stable Posm or fluid intake.

However, Hct change after the race was not associated with plasma [Na^+^] change, fluid intake or stable Posm in the present study. The change in Hct probably reflects the impact of physical extertion (Fallon et al., 1999) and the “pseudoanemia” reflects the reaction to strenuous exercise of long duration.

### Urinary Parameters, TTKG

A further important finding was that Usg and Uosm remained stable. The current evidence tends to prefer urine indices such as Usg and Uosm as the most promising indices of hydration status available, in comparison with the hematological parameters which are not as sensitive (Shirreffs, 2003). By contrast, a recent study by Hew-Butler et al. (2018) points out that Uosm and/or Usg should not be used as a surrogate measure to blood hydration markers taken at rest. Nevertheless, all finishers were euhydrated after the race according to the Usg values (Armstrong et al., 1998).

Urine [K^+^] increased and urine [Na^+^] decreased post-race; however, on the border of statistical significance. Similarly, 56-km ultra-marathoners also showed decreased urine [Na^+^] with maintained plasma [Na^+^] (Hew-Butler et al., 2008). A highly significant decrease in urine [Na^+^] was found after a 24-h endurance run (Fellmann et al., 1988) due to the effect of aldosterone. Changes in plasma and urine [K^+^] and urine [Na^+^] concentrations and increased TTKG are indirect markers of the activity of aldosterone (West et al., 1986), activation of the renin-angiotensin-aldosterone system (RAAS) and the secretion of arginine vasopressin (AVP) and they might be caused by physical exercise and sleep deprivation during the 24-h race (Fellmann et al., 1988). The AVP increases the excretion of [K^+^] in the collecting duct while promoting antidiuresis (Sonnenberg et al., 1987). The changes in electrolytes and the [K^+^] to [Na^+^] ratio < 1.0 post-race suggest that more [Na^+^] than [K^+^] was excreted during the race, however, in fact urine [K^+^] to [Na^+^] ratio increased post-race more than twice. The urine [Na^+^] losses are compatible with the syndrome of inappropriate secretion of AVP (Knechtle et al., 2012).

We suppose that aldosterone acted on the tubules in the kidneys causing them to reabsorb more [Na^+^] from the urine, thus plasma volume increased, [Na^+^] was reabsorbed to blood and [K^+^] was secreted to the tubules, where it became part of urine and was excreted. Decreased plasma [Na^+^] was partially compensated by the suppression of urine [Na^+^] excretion, similarly as in the study by Gerth et al. (2002) where aldosterone had a [Na^+^] sparing effect at the cost of [K^+^]. Plasma [Na^+^] concentration was stable, despite the increased tubular [Na^+^] reabsorption indicated by the reduction in urine [Na^+^] post-race. It seems probable that the lost urine [Na^+^] post-race was [Na^+^] stored during the race. Urine [Na^+^] reduction would then be caused by the removal of [Na^+^] conserving stimulus and homeostatic forces would restore [Na^+^] content to normal (Milledge et al., 1982). These findings suggest that the physiological mechanism responsible for body fluid homeostasis primarily preserves plasma [Na^+^] and Posm. The very prolonged nature and the distance of the 24-h race were the factors responsible for these transient changes. We conclude that adequate fluid intake enabled the parameters to return to normal levels, as Uosm and Usg remained stable.

### Limitations

This pilot observational study has several limitations worth noting. The study is limited by the overall number of subjects. The number of male subjects was not equal to the number of female subjects. It is not usual that many women participate in, volunteer and finish such a grueling mountain race in extremely cold winter conditions. The final fluid intake was calculated based on the information reported by the competitors and assistants at the aid station; it was not analyzed through dietary analysis software and water in solid food was not recorded. We also did not control the contents and composition of food and fluids during the race and at the aid station. In fact, it was impossible to capture and recognize our participants throughout the race at the single, constantly overcrowded aid station by our small research team and to monitor their intake and composition of fluids and food in detail. Nearly half of the competitors had no team support and they were not able to note their fluid intake in detail, only the overall amount during the whole 24 h. We are aware of that fact and of the possible concerns related to the associations between estimated fluid intake and plasma or urinary parameters. The absence of data on renal hormone responses, urine losses, metabolic water gain, respiratory water, sweat losses, and runners’ body temperature may also limit the present study. The extremely adverse weather conditions and the difficulty of this race prevented some measurements and more detailed records. On the other hand, the study includes a relatively high number of ultra-endurance female finishers and it is the first study of ultra-marathoners competing in such extremely cold winter conditions.

## Conclusion

Hydration status remained stable despite the extremely cold winter weather conditions. The overall fluid intake was probably appropriate to replenish the hydration needs of the 24-h runners. Current recommendation may be too high for athletes competing in extremely cold conditions.

## Author Contributions

DC and BK designed the study and collected the data. DC, PN, TR, BK, and JB contributed by writing and editing a part of the manuscript and contributed by reviewing and editing the manuscript. All authors have read and approved the final manuscript.

## Conflict of Interest Statement

The authors declare that the research was conducted in the absence of any commercial or financial relationships that could be construed as a potential conflict of interest.
